# Relationship between vitamin D and surrogate parameters (leukocyte telomerase length, telomerase activity), and genes (*ACTN3, FOXO3A, VDR, SIRT1, MSTN*) for ageing in Asian Indian with prediabetes

**DOI:** 10.3389/fendo.2026.1740628

**Published:** 2026-03-24

**Authors:** Surya Prakash Bhatt, Amisha Khurana, Shivam Pandey, Anoop Misra

**Affiliations:** 1 Fortis Centre of Excellence for Diabetes, Metabolic Diseases, and Endocrinology, Chirag Enclave, New Delhi, India; 2 National Diabetes, Obesity and Cholesterol Foundation (N-DOC), Safdarjung Development Area, New Delhi, India; 3 Department of Medicine, All India Institute of Medical Sciences, New Delhi, India; 4 Diabetes Foundation (India), SDA, New Delhi, India; 5 Department of Biostatistics, All India Institute of Medical Sciences, New Delhi, India

**Keywords:** ACTN3, FOXO3A, leukocyte telomere length, MSTN, SIRT1, telomerase activity, VDR

## Abstract

**Introduction:**

Vitamin D has been implicated in cellular aging through its effects on telomere maintenance, but the extent of its influence on leukocyte telomere length (LTL), telomerase activity (TA), and interactions with aging-related genetic polymorphisms remains underexplored in Asian Indians with prediabetes. This study investigated the relationship between serum vitamin D levels, LTL, TA, and variants in aging-associated genes—*ACTN3, FOXO3A, VDR, SIRT1, and MSTN.*

**Methods:**

A cross-sectional study was conducted among 290 prediabetic subjects (aged 20–60 years). Anthropometry, biochemical markers, LTL (by qPCR), TA (by TRAP assay), and genotyping (PCR-RFLP) were assessed. Regression analyses were used to determine independent associations, adjusted for age, sex, BMI, and fasting blood glucose. Gene-gene interactions were evaluated *via* pearson correlation.

**Results:**

Vitamin D levels showed a statistically significant but modest positive correlation with LTL (p = 0.03), which attenuated after adjustment (p = 0.06). No significant association was found between vitamin D and TA (p = 0.26). The ACTN3 polymorphism was significantly associated with TA (adjusted B = 0.10, p = 0.03). Strong gene-gene interactions were observed among SIRT1, MSTN, VDR, and FOXO3A (r = 0.56–0.82; p < 0.001), indicating a coordinated genetic influence on aging and metabolism.

**Discussion:**

In Asian Indian prediabetic individuals, vitamin D exhibits only modest association with LTL, whereas a coordinated network of aging-related genes appears to exert a more substantial influence on cellular aging and metabolic risk. These preliminary findings suggest potential gene-vitamin D interactions warranting longitudinal and interventional studies for precision approaches in prediabetes management.

## Introduction

The global prevalence of prediabetes continues to rise, with approximately 10-14% of the adult population in India estimated to have this condition (1, 2). Prediabetes represents a critical phase in the continuum leading to type 2 diabetes and is increasingly recognized as a state associated with accelerated biological aging (3). Despite this growing burden, the relationship between prediabetes and cellular aging has received limited research attention, particularly in high-risk populations such as Asian Indians.

Cellular aging is closely linked to telomere biology. Telomeres are protective structures at the ends of chromosomes that shorten with each cell division, eventually leading to cellular senescence and increased susceptibility to chronic diseases (4). Leukocyte telomere length (LTL) serves as an important surrogate marker of cellular aging (5). Telomerase, the enzyme responsible for maintaining telomere length, counteracts this shortening process, though its activity is often diminished in the context of aging and metabolic disorders (6). Understanding the factors that influence telomere biology may provide insights into strategies to slow biological aging and prevent age-related diseases.

Metabolic dysregulation increases biological aging by increasing telomere attrition. Individuals with metabolic abnormalities (hyperglycemia, insulin resistance, dyslipidemia, obesity, hypertension) have shorter LTL (7). Chronic low grade inflammation, increased oxidative stress damage DNA at the telomeric region (8) and impaired glucose and lipid metabolism (9) lead to ageing of the cellular structures, including telomeres. Specifically, pro-inflammatory cytokines such as IL-6 and TNF-α suppress telomerase activity and lead to telomere shortening (10).

Vitamin D is directly linked with pancreatic β-cell function. β-cells have both vitamin D receptors (VDR) as well as enzyme 1-α-hydroxylase. Latter enzyme converts 25-hydroxyvitamin D into its active form. The active metabolite 1,25-dihydroxyvitamin D can enhance insulin production. It interacts with VDR and modulates transcription of the insulin gene. It also affects the calcium dynamics intra-cellularly a key driver of glucose-stimulated insulin release, thereby improving the secretory function of β-cells (11).

Beyond its metabolic effects, emerging evidence suggests that vitamin D may influence the expression and function of several longevity-associated genes. Alpha-actinin-3 (ACTN3) is associated with muscle function and has been linked to physical performance and longevity (12). Forkhead Box O3A (FOXO3A), a well-established longevity gene, regulates cellular stress responses and has been associated with increased lifespan across various populations (13). The following two genes affect critical determinants of ageing including insulin sensitivity, muscle health and metabolic status. The vitamin D receptor (VDR) gene itself is critical for mediating vitamin D’s effects on gene expression and cellular processes, including insulin sensitivity (14). Sirtuin 1 (SIRT1) is involved in cellular regulation, influencing metabolic processes and lifespan (15), while Myostatin (MSTN) regulates muscle mass with implications for metabolic health (16). Our previous research demonstrated that variants of the Myostatin gene (A55T and K153R polymorphisms) are associated with increased obesity, abdominal obesity, and lower lean body mass in non-diabetic Asian Indians in North India (17).

In our study, we deliberately focused on individuals with prediabetes. Prediabetes represents a critical phase in the continuum leading to diabetes and accelerated biological ageing (3). Studying this group allows us to capture early pathophysiological changes before the onset of overt disease. An additional advantage of selecting individuals with prediabetes is methodological. In this stage, it is easier to exclude or restrict the use of drugs that could influence LTL or TA, such as metformin. This helps ensure a more homogeneous study population that is largely free from the confounding effects of pharmacotherapy.

Importantly, most existing evidence linking vitamin D status with telomere biology comes from Western populations, often involving healthy adults. Data from Asian Indians are scarce, despite this population having a high prevalence of vitamin D deficiency (18), metabolic disorders, and features of accelerated ageing (19). Finally, our own earlier cross-sectional work on LTL and TA was conducted in individuals with prediabetes (20). Building on this prior experience and data set gives us greater confidence in selecting this subgroup for the present investigation”.

This study aims to explore the relationship between vitamin D levels, LTL, TA, and aging-related genes (ACTN3, FOXO3A, VDR, SIRT1, MSTN) in subjects with prediabetes. By analyzing the interplay among these factors, the study hoped to understand any significant associations. This investigation may reveal novel preventive and therapeutic targets for age-related metabolic deterioration in a population with unique genetic and environmental factors affecting vitamin D metabolism, aging mechanisms, and diabetes progression.

## Materials and methods

### Study design and subjects recruitment

**Figure 1:**
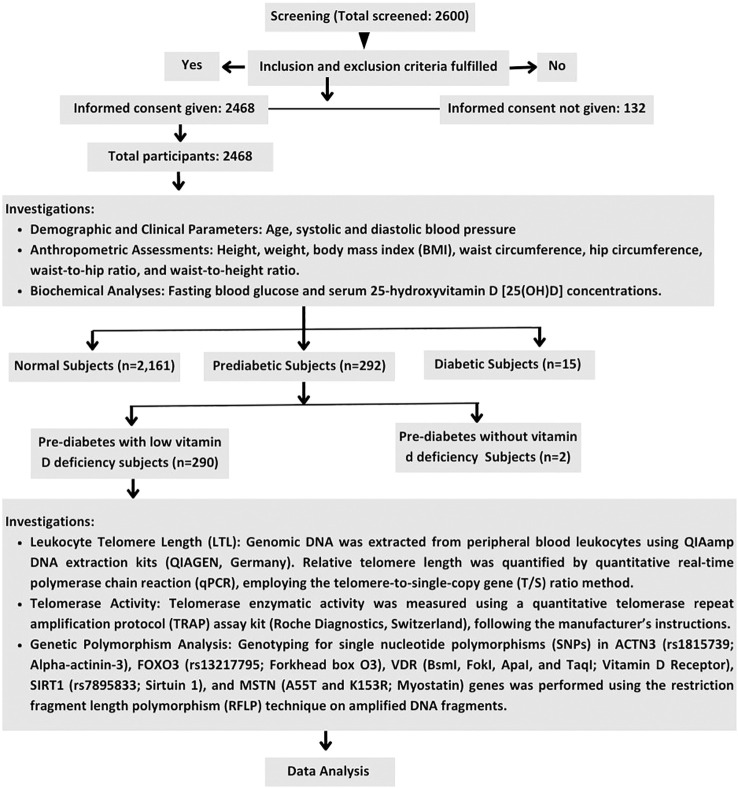
Study flow chart. This flowchart shows the participant selection process for a diabetes genetics study. Of 2,600 individuals screened, 2,468 provided informed consent and were included. Participants were categorized into normal (n=2,161), prediabetic (n=292), and diabetic (n=15) groups based on standard clinical criteria.

**Figure 2:**
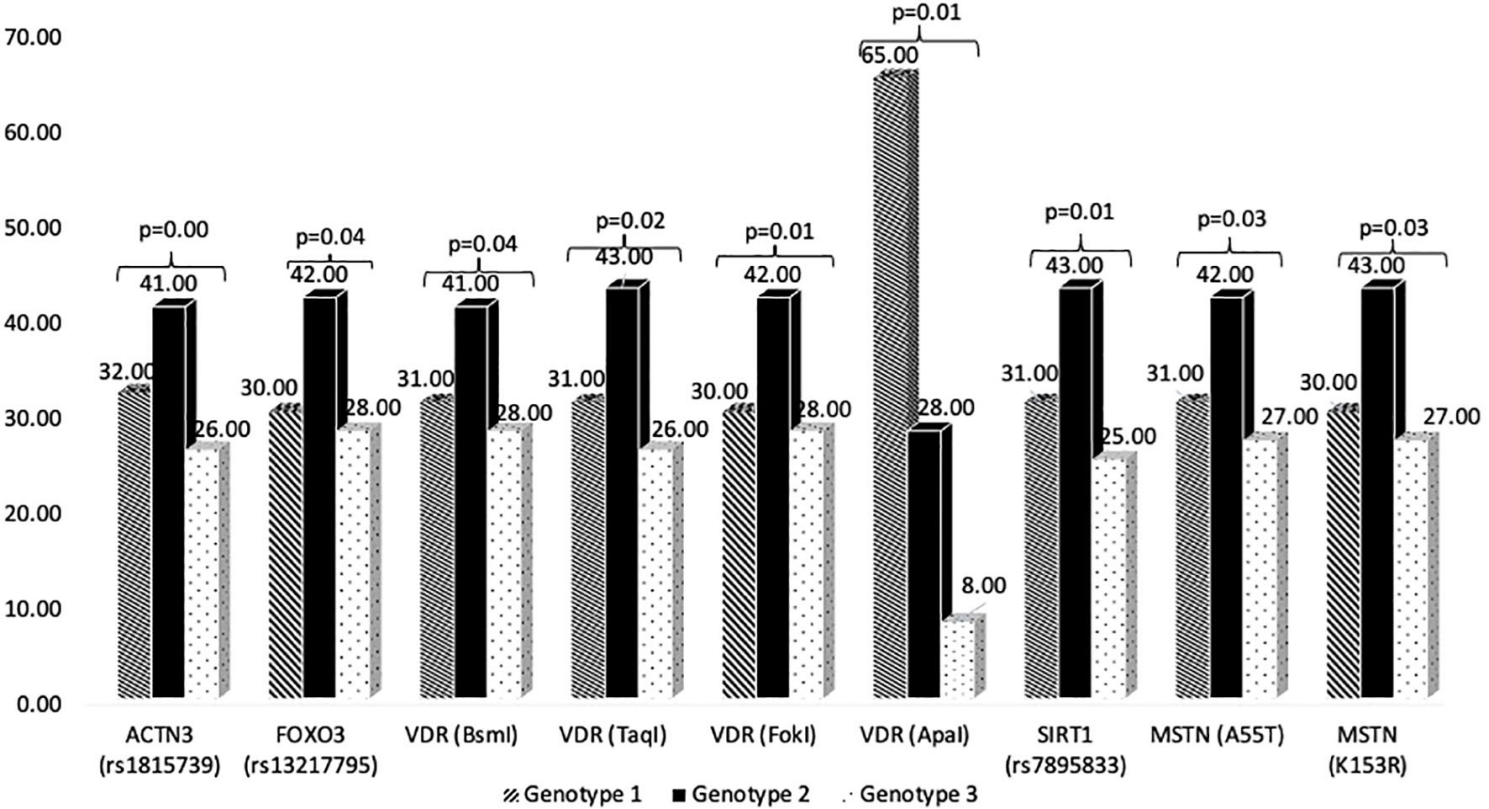
Genotype distribution. This bar chart displays genotype frequencies for eight genetic polymorphisms across ACTN3, FOXO3, VDR, SIRT1, and MSTN genes. Each gene shows three genotype variants with statistical comparisons. VDR (ApaI) shows the most dramatic difference (p=0.01) with one genotype at 65% prevalence versus 28% and 8% for others. Several other polymorphisms show significant distributions (p-values 0.01-0.04), suggesting these genetic variants may influence diabetes risk or metabolic traits in the study population.

This study was designed as a cross-sectional population-based analysis conducted from July 2020 to December 2024, aimed at evaluating various physiological, biochemical, and genetic markers in individuals diagnosed with prediabetes. Of 2,468 subjects screened (**Figure 1**), 2, 161 were classified as normal, 15 as diabetic, and 292 as prediabetic, with the final data analysis conducted on 290 prediabetic subjects (aged 20–60 years) with vitamin D deficiency who had fasting plasma glucose concentrations between 100 and 125 mg/dL. Ethical clearance was obtained from Fortis C-DOC Centre of Excellence for Diabetes, Metabolic Diseases & Endocrinology, New Delhi, India, and informed written consent was secured from all participants. Study subjects were randomly designated to ensure approximate representation from each income group, while exclusion criteria included: individuals who had received vitamin D or calcium supplementation in the last six months, those on medications influencing insulin secretion, insulin sensitivity, or vitamin D and calcium metabolism within the past month, those with severe end-organ damage or chronic diseases (e.g., renal or hepatic failure, malignancies, major systemic illnesses), and known cases of diabetes, HIV infection, or other endocrine disorders.

### Demographic, clinical profiles, anthropometry, and biochemical analysis

Demographic and clinical profiles were assessed using a validated questionnaire. Body mass index (BMI), waist circumference (WC), hip circumference (HC) were recorded (21). In addition, we have categorized the subjects according to the BMI and found 29.73% was normal BMI, 22.29% was overweight and was 47.98% was obese subjects. Ratios such as waist-hip ratio (WHR) and waist-height ratio (WhtR) were calculated. Venous blood samples were collected and processed for fasting blood glucose (FBG) and serum 25-hydroxy vitamin D [25(OH)D] levels, as previously described by Bhatt et al. (21).

### Gene polymorphisms

Fasting blood samples (5 ml) were collected in tubes containing EDTA for genetic analysis. Genomic DNA was extracted using the QIAamp Blood Kit (QIAGEN) and amplified using polymerase chain reaction (PCR). Single nucleotide polymorphisms (SNPs) in candidate genes related to aging (*ACTN3, SIRT1, FOXO3A, VDR, MSTN)* were analyzed using PCR-restriction fragment length polymorphism (RFLP) analysis (12, 15, 17, 22, 23).

### Measurement of leukocyte telomerase length and telomerase activity

DNA was extracted from peripheral blood mononuclear cells and quantified for further experiments (6). Relative LTL in genomic DNA extracted from peripheral blood leukocytes was measured using quantitative real-time polymerase chain reaction (qPCR), with the telomerase repeat copy number (T) compared to a single-copy gene (S) to generate a T/S ratio (6, 24). Telomerase activity was assessed using a telomerase assay kit (Roche, Switzerland) (25).

### Gen gene interaction analysis

Gene–gene interaction was assessed by evaluating the pairwise correlations among the genotyped variants, including *ACTN3, FOXO3, VDR (BsmI and TaqI), SIRT1, and MSTN (A55T and K153R)*. Genotype data were coded numerically based on allele count using an additive genetic model (0=homozygous wild-type, 1=heterozygous, 2=homozygous variant). The correlation between the genetic variants was done by Pearson’s correlation. Significance was assessed using two-tailed p-values, with p<0.05 considered statistically significant. All statistical analysis was performed using SPSS Software and STATA. A correlation matrix was generated, and results were visualized using a grayscale heatmap to highlight the magnitude and direction of associations among gene variants. The heatmap displays correlation strength on a scale from 0.00 to 1.00, with darker shading indicating stronger positive correlations.

### Statistical analysis

Data was compiled in an Excel spreadsheet (Microsoft, Redmond, Washington, USA), where we assessed the distribution of demographic, clinical, socioeconomic, medical history (both personal and familial), behavioral traits, sun exposure, skin exposure, and biochemical test results for normality. Descriptive statistics, including mean, standard deviation (SD), and frequency counts (number and percentage), were utilized to summarize the dataset.

To investigate the correlation between LTL and various measures of body composition, we conducted Pearson correlation analyses. The impact of genetic variants and vitamin D levels on LTL was evaluated using linear regression models. We provided both unadjusted and adjusted estimates, with adjustments made for potential confounding variables such as age, sex, BMI, and other pertinent clinical parameters. Each variable’s association with LTL was reported as unstandardized coefficients (B) along with their corresponding 95% confidence intervals to assess the precision of these estimates.

To gauge the overall fit of the regression models, we computed R-squared values, which indicate the proportion of variance in LTL explained by the genetic and vitamin D factors. Additionally, we performed independent samples t-tests to compare LTL and activity between males and females, and employed one-way analysis of variance (ANOVA) to assess differences across three age groups (20-30, 31-40, and 41–50 years). All statistical analyses were carried out using STATA version 16.0 (StataCorp LLC, College Station, TX, USA), and a significance level of p < 0.05 was considered for all tests.

## Results

### Demographic clinical, anthropometry and biochemical profile

Demographic, clinical, anthropometric and biochemical investigations of 2468 subjects have been shown in **Supplementary Table 1**. Male subjects were significantly more (63.71%) as compared to male subjects (36.29%). Mean age, BMI, WC, HC, WHR, W-htR, FBG and vitamin D levels was shown in **Supplementary Table 1**.

Analysis of LTL (n=290 prediabetes subjects) revealed no statistically significant differences between males and females (p = 0.12), nor among age groups (20–30 years; 31–40 years; 41–50 years: p = 0.10). Similarly, telomerase activity did not differ significantly between males and females, and remained consistent across all age categories (20–30 years; 31–40 years; 41–50 years; p = 0.96). Results showed no significant correlation between Vitamin D levels and BMI (**Supplementary Table 2**).

### Vitamin D and its correlation and regression analysis

Analysis revealed vitamin D showed a small but significant positive correlation with LTL (r = 0.11, p = 0.03), but no relationship with telomerase activity (r = -0.06, p = 0.26). FBG correlated positively with TA (r = 0.18, p = 0.0007), and height with LTL (r = 0.14, p = 0.006).

Regression analysis revealed a statistically significant positive association between Vitamin D levels and LTL (p = 0.03; 95% CI: 1.10 [44.05]). Associations with aging-related genes (*ACTN3, FOXO3A, VDR, SIRT1, MSTN*) were not statistically significant (p > 0.05), and telomerase activity showed no significant association with vitamin D (p = 0.26) (**Table 1**).

**Table 1:** Regression of Vitamin D on aging genes, leukocyte telomerase length and activity.

Variables	p	95% CI
Alpha -actinin-3 (rs1815739)	0.10	-4.54(0.40)
Forkhead Box O3A (rs13217795	0.17	-4.19(0.74)
Vitamin D receptor (Bsml)	0.08	-4.58(0.32)
Vitamin D receptor (Taql)	0.11	-4.51(0.46)
Sirtuin -1 (rs7895833)	0.43	-3.50(1.51)
Myostatin (K153R)	0.39	-3.58(1.41)
Leucocyte Telomerase length	0.03	1.10(44.05)
Telomerase activity	0.26	-12.6(3.48)

p, significance level; 95% CI, confidence interval with standard error in parentheses. ACTN3, alpha-actinin-3 gene; FOXO3A, longevity gene; VDR, vitamin D receptor; SIRT1, sirtuin-1 aging gene; MSTN, myostatin muscle gene. Leukocyte telomerase length significantly associated (p=0.03).

### Single nucleotide polymorphisms of aging genes in subjects with prediabetes

ACTN3 alleles: R (43.38%), X (56.62%); X/X genotype (27.54%) linked to higher BMI, abdominal obesity, and adverse lipid profiles. FOXO3A genotypes: CC (30.45%), C/T (41.92%), TT (27.54%) (p=0.0358). SIRT1 genotypes: GG (31.14%), GA (43.41%), AA (25.45%). MSTN variants A55T and K153R showed predominant heterozygosity (41.92% and 41.02%, respectively). VDR BsmI b/b genotype (27.54%) associated with higher BMI and abdominal obesity (n=290, **Supplementary Tables 3**, **Figure 2**).

### Associations between gene variants, vitamin D levels, LTL, and telomerase activity

**Figure 3:**
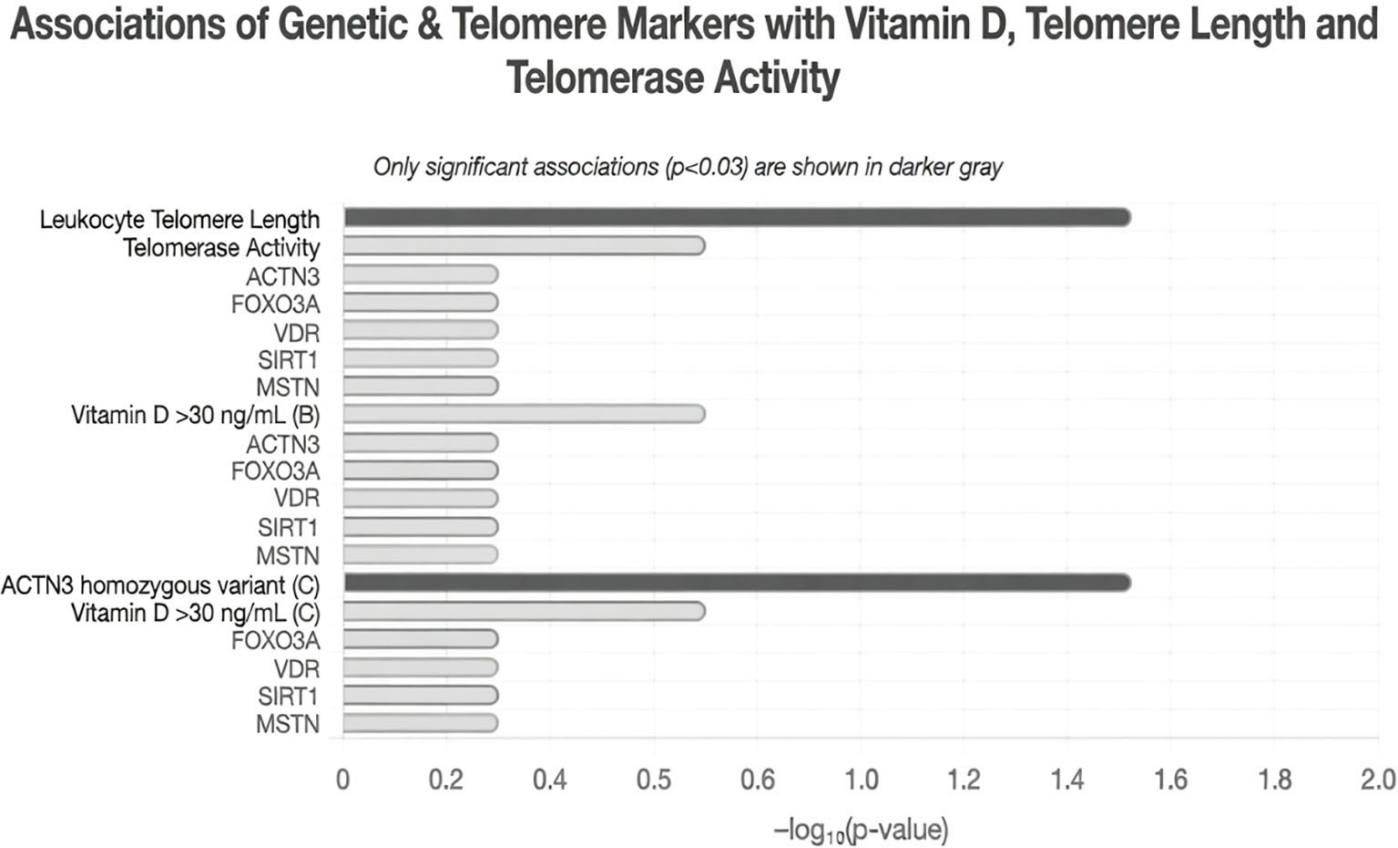
Associations of selected genetic markers and vitamin D status with leukocyte telomere length and telomerase activity. This figure displays the strength of associations (as –log_10_ p-value) between selected genetic markers (ACTN3, FOXO3A, VDR, SIRT1, MSTN), the ACTN3 homozygous variant, and vitamin D status (>30 ng/mL) with leukocyte telomere length and telomerase activity. Only statistically significant associations (p < 0.03) are shown in darker gray bars; longer bars indicate stronger significance. (B) = vitamin D threshold in the overall group; (C) = vitamin D threshold specifically in ACTN3 homozygous variant carriers.

Linear regression analysis examining associations between gene variants, vitamin D levels, and LTL revealed few significant relationships. ACTN-3 showed a potential association in the unadjusted model (p=0.008), but this weakened after adjustment (p=0.09). Vitamin D levels above 30 ng/mL showed a statistically significant association with LTL in the unadjusted model (p=0.03), though this relationship was attenuated in the adjusted model (p=0.06). For telomerase activity, only ACTN-3 showed a significant positive association in the third category (adjusted B = 0.10, p = 0.03) (**Table 2**, **3**; **Figure 3**).

**Table 2:** Linear regression of genes and Vitamin D on telomerase length.

Variable	Unadjusted B	p-value	Adjusted B (95% Confidence Interval)	p-value
ACTN3 (rs1815739)	1	Reference		0.09
	2	0.008	0.44 (-0.004, -0.01)	0.09
	3	0.003	0.80 (-0.01, 0.06)	0.17
FOXO3 (rs13217795)	1	Reference		0.96
	2	0.97	0.001 (-0.04, 0.04)	0.96
	3	-0.01	0.35 (-0.07, 0.02)	0.39
VDR (BsmI)	1	Reference		0.54
	2	-0.00	0.63 (-0.05, 0.03)	0.54
	3	-0.00	0.47 (0.01, -0.03)	0.64
VDR (TaqI)	1	Reference		0.23
	2	-0.00	0.72 (-0.07, 0.01)	0.23
	3	-0.01	0.18 (-0.05, -0.10)	0.06
SIRT1 (rs7895833)	1	Reference		0.46
	2	0.00	0.84 (-0.02, 0.05)	0.46
	3	-0.00	0.67 (-0.02, 0.08)	0.24
MSTN (A55T)	1	Reference		0.19
	2	-0.01	0.36 (-0.06, 0.01)	0.19
	3	-0.01	0.38 (-0.04, 0.04)	0.95
MSTN (K153R)	1	Reference		0.23
	2	0.00	0.76 (-0.01, 0.07)	0.23
	3	-0.00	0.60 (-0.05, 0.05)	0.93
Vitamin D (>30)	Reference	Reference		0.06

Genotypes coded as 1=reference, 2=heterozygous, 3=homozygous variant. Adjusted B = coefficient after controlling for covariates. Vitamin D >30 ng/mL = sufficient levels. Most associations non-significant.

**Table 3:** Linear regression analysis of various genes and vitamin D on telomerase activity.

Variable	Unadjusted B	p-value	Adjusted B (95% Confidence Interval)	p-value
Alpha -actinin-3 (rs1815739)	1	Reference		0.94
	2	0.00	-0.01 (-0.01, 0.07)	0.72
	3	0.07	0.10 (0.00, 0.20)	0.03*
Forkhead Box O3A (rs13217795)	1	Reference		0.64
	2	0.01	0.00 (-0.12, 0.13)	0.91
	3	0.01	-0.09 (-0.22, 0.03)	0.16
Vitamin D Receptor (BsmI)	1	Reference		0.86
	2	-0.00	-0.03 (-0.15, 0.08)	0.54
	3	0.03	0.05 (-0.06, 0.17)	0.39
Vitamin D Receptor (TaqI)	1	Reference		0.37
	2	0.02	0.05 (-0.06, 0.18)	0.35
	3	0.05	0.07 (-0.06, 0.21)	0.30
Sirtuin-1(rs7895833)	1	Reference		0.78
	2	0.00	0.01 (-0.09, 0.12)	0.84
	3	0.01	0.00 (-0.14, 0.15)	0.94
Myostatin (A55T)	1	Reference		0.81
	2	-0.00	-0.05 (-0.15, 0.04)	0.27
	3	0.02	-0.01 (-0.12, 0.09)	0.80
Myostatin (K153R)	1	Reference		0.36
	2	0.02	0.02 (-0.09, 0.14)	0.71
	3	0.02	-0.07 (-0.22, 0.06)	0.28
Vitamin D (>30 ng/mL)	0 (>30)	Reference		0.26
	1 (>30)	-0.00	-0.00 (-0.00, 0.00)	0.49

Genotypes coded as 1=reference, 2=heterozygous, 3=homozygous variant. *p<0.05 indicates significance. Only ACTN3 genotype 3 significantly associated with telomerase activity (p=0.03). All other genetic variants show minimal effects.

### Gene-gene interaction and its implications in muscle function, metabolism, and aging

**Figure 4:**
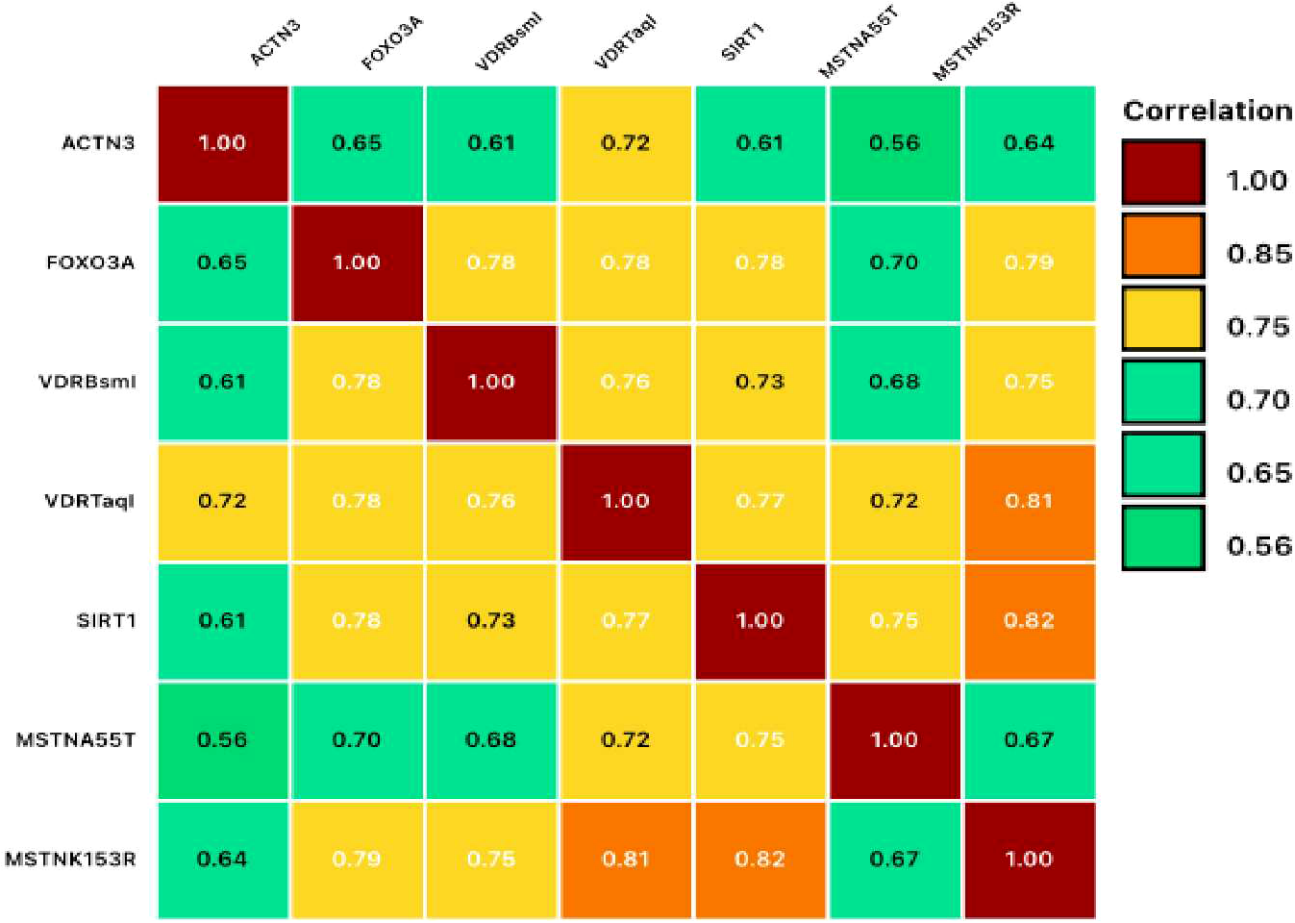
Gene-gene interaction heatmap. This correlation matrix reveals moderate to strong positive correlations (0.56-0.82) between different genetic polymorphisms. The strongest associations include MSTN K153R with VDR TaqI (0.82), SIRT1 with VDR TaqI (0.81), and FOXO3 with VDR BsmI (0.78). These correlations suggest interconnected genetic networks that may collectively influence metabolic pathways, aging processes, or diabetes susceptibility through shared biological mechanisms.

Gene-gene interaction analysis revealed significant correlations among genetic variants, including ACTN3, FOXO3, VDR (BsmI, TaqI), SIRT1, and MSTN (A55T, K153R), with strong associations (r = 0.56–0.82, p = 0.00) (**Table 4**). Notably, SIRT1 showed a strong correlation with MSTN (K153R) and VDR (TaqI), suggesting a link between metabolic and muscle-related pathways. FOXO3 was consistently correlated with VDR and SIRT1, highlighting its role in longevity and metabolic regulation (**Figure 4**).

**Table 4:** Multi-dimensional analysis of gene-gene interactions.

Primary gene	Interacting gene	Interaction coefficient
Alpha -actinin-3 (rs1815739)	FOXO3 (Ars13217795)	0.65 (p<0.001)
	VDR (BsmI)	0.61 (p<0.001)
	VDR (TaqI)	0.72 (p<0.001)
	SIRT1 (rs7895833)	0.61 (p<0.001)
	MSTN (A55T)	0.56 (p<0.001)
	MSTN (K153R)	0.64 (p<0.001)
Forkhead Box O3A (rs13217795)	VDR (BsmI)	0.78 (p<0.001)
	VDR (TaqI)	0.78 (p<0.001)
	SIRT1 (rs7895833)	0.78 (p<0.001)
	MSTN (A55T)	0.70 (p<0.001)
	MSTN (K153R)	0.79 (p<0.001)
Vitamin D receptor (BsmI)	VDR (TaqI)	0.76 (p<0.001)
	SIRT1 (rs7895833)	0.73 (p<0.001)
Myostatin (A55T)	MSTN (A55T)	0.68 (p<0.001)
	MSTN (K153R)	0.75 (p<0.001)
Vitamin D receptor (TaqI)	SIRT1 (rs7895833)	0.77 (p<0.001)
	MSTN (A55T)	0.72 (p<0.001)
	MSTN (K153R)	0.81 (p<0.001)
Sirtuin -1 (rs7895833)	MSTN (A55T)	0.75 (p<0.001)
	MSTN (K153R)	0.82 (p<0.001)
Myostatin (K153R)	MSTN (K153R)	0.67 (p<0.001)

All interaction coefficients highly significant (p<0.001). Values range 0.56-0.82 indicating moderate to strong gene-gene correlations. Strongest interaction: SIRT1-MSTN(K153R) = 0.82.

## Discussion

In this study, we observed a positive correlation between vitamin D levels and LTL that attenuated after adjustment for age, sex, BMI, and FBG, while no association was found between vitamin D and TA. Among genetic variants examined, only ACTN3 homozygous variant demonstrated significant association with increased TA. Notably, we identified strong gene-gene interactions among ACTN3, FOXO3A, VDR, SIRT1, and MSTN.

The attenuated vitamin D-LTL association suggests limited direct effects on telomere biology in prediabetes in this in this sample of population. A previous study demonstrated that vitamin D supplementation attenuated telomere shortening in individuals with metabolic syndrome (26). Similarly, another study has reported that vitamin D sufficiency was associated with longer LTL in a longitudinal study of prediabetic individuals transitioning to diabetes (27). While we (longitudinal study done in women) (18) and others (20) have shown increase LTL and TA after vitamin D supplementation but cross sectional nature of current study limits robust conclusion. Further, in our study, most genetic variants showed no independent associations with telomere parameters after adjustment, with ACTN3 as the notable exception. Previous studies have suggested that ACTN3 may influence muscle damage susceptibility and recovery processes, which could theoretically impact cellular senescence pathways relevant to telomere dynamics (28). In our study, ACTN3 homozygous variant carriers exhibited 20% higher TA, but no similar data are available from literature. Unlike our findings, Willcox et al. demonstrated that FOXO3A genetic variation was strongly associated with human longevity, with homozygous minor allele carriers showing 2.75-fold increased odds of longevity. Favorable FOXO3A genotypes were linked to healthy aging phenotypes including enhanced insulin sensitivity, lower disease prevalence, and superior physical and cognitive function (29). Strong gene–gene interactions observed among ACTN3, FOXO3A, VDR, SIRT1, and MSTN in our study warrants further discussion. Such interactions suggest presence of integrated biological networks linking mitochondrial regulation, muscle metabolism, insulin signaling, and vitamin D–mediated transcriptional control.

A key aim of aging research is to identify genetic targets to extend lifespan, but most longevity genes from animal models fail to replicate in humans. This may be due to the conditional and heterogeneous nature of genetic effects, driven by gene–gene and gene–environment interactions rather than single genes. Ukraintseva et al. show that epistatic interactions among aging-pathway genes (IGF1/FOXO, p53/p16, mTOR) are significantly associated with survival beyond 85 years in humans (30)). Like findings in human longevity research, studies related to age-related macular degeneration (AMD) illustrate how complex age-associated diseases are driven by multiple genes and their interactions rather than single genetic variants. Using a forest-based analytical approach, this study demonstrates that gene–gene and gene–environment interactions uncover both known and novel high-risk haplotypes associated with AMD (31). Evidence from genome-wide studies of exceptional longevity further supports the concept that complex aging phenotypes arise from polygenic architectures and genetic signatures rather than single variants. A large genome wide association study (GWAS) of centenarians demonstrated that a model incorporating 281 SNPs could accurately classify exceptional longevity, with predictive power increasing at more extreme ages. Notably, removal of the strongest single signal (TOMM40/APOE) minimally reduced model performance, underscoring the dominance of cumulative and interacting genetic effects (32). The identification of distinct longevity “genetic signatures” mirrors findings from gene–gene interaction studies, reinforcing that aging and survival are best explained through network-based, multi-locus models rather than isolated genes.

This study has several important limitations. The cross-sectional design precludes causal inference and cannot establish temporal relationships between vitamin D deficiency, genetic variants, and telomere dynamics. The modest sample size (n=290) may have limited power for genotype-stratified analyses, the candidate gene approach examined only five genes, and the homogeneous Asian Indian population limits generalizability to other ethnic groups. The absence of vitamin D-sufficient prediabetic controls and vitamin D-deficient non-prediabetic controls prevents determination of whether associations are specific to prediabetes or represent general vitamin D deficiency effects. Peripheral blood leukocyte measurements may not reflect aging processes in metabolically active tissues such as pancreatic β-cells, skeletal muscle, or adipose tissue. Important confounding factors including oxidative stress biomarkers, inflammatory cytokines, dietary intake, sunlight exposure, physical activity, and psychosocial stress were not systematically measured.

Future research should include longitudinal cohort studies with serial measurements to establish temporal relationships and randomized controlled trials of vitamin D supplementation to determine causality in genetically stratified prediabetic populations. Studies should incorporate appropriate control groups (vitamin D-sufficient prediabetics and vitamin D-deficient non-prediabetics), tissue-specific telomere analyses, and comprehensive assessment of oxidative stress, inflammation, dietary factors, and lifestyle confounders. Multi-ethnic cohorts are needed to examine population-specific effects and gene-environment interactions across diverse genetic backgrounds. Genome-wide association studies may identify novel genetic variants beyond our candidate genes, and mechanistic studies using cellular and animal models would provide biological validation and identify therapeutic targets for precision medicine approaches in prediabetes management.

## Conclusion

In this cross-sectional analysis, vitamin D showed at most a modest association with LTL and no association with TA, while notable correlations were observed among several aging-related genes in individuals with prediabetes. These findings provide preliminary insights into the interplay between vitamin D status, telomere biology, and genetic networks. However, given the study design and effect sizes, the results should be interpreted with caution. Well-designed longitudinal studies and randomized trials are required to confirm these observations and clarify their clinical relevance.

## Data Availability

The datasets presented in this study can be found in online repositories. The names of the repository/repositories and accession number(s) can be found in the article/**Supplementary Material**.
