# Supplementary material for: Relationship between vitamin D and surrogate parameters (leukocyte telomerase length, telomerase activity), and genes (ACTN3, FOXO3A, VDR, SIRT1, MSTN) for ageing in Asian Indian with prediabetes
Source: Front Endocrinol (Lausanne). 2026 Mar 24;17:1740628. doi: 10.3389/fendo.2026.1740628 (PMC13053265; doi:10.3389/fendo.2026.1740628)
Supplement: Supplementary file 1 [file Supplementaryfile1.docx]

**Supplementary table 1**: Demographic, clinical, anthropometric and biochemical investigations

| **Variables** | **Mean ± SD (n=2468)** | **95% CI** |
| --- | --- | --- |
| Age (Years) | 34.72 ± 8.07 | 30.40 - 39.27 |
| Systolic Blood Pressure (mmHg) | 119.81 ± 17.51 | 112.2 - 135.51 |
| Diastolic Blood Pressure (mmHg) | 84.3 ± 10.62 | 76.91 - 89.87 |
| Height (cm) | 162.44 ± 8.47 | 151.95 - 168.77 |
| Weight (Kg) | 66.63 ± 12.45 | 61.29 - 78.32 |
| Body Mass Index (Kg/m²) | 25.23 ± 4.30 | 22.3 - 30.23 |
| Waist Circumference (cm) | 89.77 ± 9.60 | 71.23 - 115.6 |
| Hip Circumference (cm) | 96.77 ± 6.92 | 75.62 - 115.18 |
| Waist-Hip Ratio | 0.93 ± 0.09 | 0.79 - 0.99 |
| Waist-Height Ratio | 0.57 ± 0.10 | 0.51 - 0.64 |
| Fasting Blood Glucose (mg/dL) | 110 ± 7.45 | 63.25 - 124.65 |
| Vitamin D (ng/mL) | 27.05 ± 15.40 | 10.23 - 32.33 |

**Supplementary table 2**: Comparison of leukocyte telomerase length and activity by gender and age group.

| Gender and Telomerase Length (n, 292) | | |
| --- | --- | --- |
| Males | 0.87±0.005 | 0.12 |
| Females | 0.86±0.007 |  |
| Gender and Telomerase Activity (n,292) | | |
| Males | 1.53±0.23 | 0.18 |
| Females | 1.49±0.01 |  |
| Age and Telomerase Length | | |
| 20-30 years | 0.85±0.09 | 0.10 |
| 31-40 years | 0.88±0.08 |  |
| 41-50 years | 0.86±0.09 |  |
| Age and Telomerase Activity | | |
| 20-30 years | 1.52±0.24 | 0.96 |
| 31-40 years | 1.51±0.24 |  |
| 41-50 years | 1.51±0.22 |  |

**Supplementary Table 3: Frequency and percentage of various genes**

| **Genes** | **Genotype 1** | **Genotype 2** | **Genotype 3** | **p-value** |
| --- | --- | --- | --- | --- |
| ACTN3 (rs1815739) | 108 (32.34%) | 140 (41.92%) | 86 (25.75%) | 0.0012 |
| FOXO3 (rs13217795) | 102 (30.45%) | 140 (41.92%) | 92 (27.54%) | 0.0358 |
| VDR (BsmI) | 104 (31.14%) | 138 (41.32%) | 92 (27.54%) | 0.0427 |
| VDR (TaqI) | 103 (30.84%) | 143 (42.81%) | 88 (26.35%) | 0.0241 |
| VDR (FokI) | 100 (30.00%) | 140 (42.00%) | 94 (28.00%) | 0.0123 |
| VDR (ApaI) | 107 (64.50%) | 46 (27.50%) | 13 (8.00%) | 0.0123 |
| SIRT1 (rs7895833) | 104 (31.14%) | 145 (43.41%) | 85 (25.45%) | 0.0195 |
| MSTN (A55T) | 103 (30.84%) | 140 (41.92%) | 91 (27.25%) | 0.0312 |
| MSTN (K153R) | 100 (29.94%) | 144 (43.11%) | 90 (26.95%) | 0.0269 |

**Supplementary table 4: Association of ACTN3 (rs1815739) with the following variables**

| **Variables** | **Genotype 1** | **Genotype 2** | **Genotype 3** | **p-value** |
| --- | --- | --- | --- | --- |
| Height (cm) | 161.99 ± 7.64 | 160.83 ± 7.99 | 163.33 ± 8.36 | 0.07 |
| Weight (kg) | 70.47 ± 13.29 | 70.97 ± 13.25 | 72.81 ± 11.84 | 0.42 |
| BMI (kg/m²) | 26.79 ± 4.36 | 27.41 ± 4.60 | 27.28 ± 4.02 | 0.53 |
| Waist Circumference (cm) | 96.94 ± 8.06 | 95.75 ± 9.87 | 96.13 ± 7.79 | 0.56 |
| Hip Circumference (cm) | 98.71 ± 6.92 | 99.05 ± 7.38 | 97.22 ± 6.73 | 0.15 |
| Waist-to-Hip Ratio (WHR) | 0.59 ± 0.05 | 0.59 ± 0.06 | 0.58 ± 0.05 | 0.50 |
| Waist-to-Height Ratio (WhRT) | 0.97 ± 0.06 | 0.96 ± 0.07 | 0.98 ± 0.06 | 0.02* |
| Fasting Blood Glucose (mg/dL) | 110.57 ± 7.94 | 110.58 ± 7.51 | 110.46 ± 7.75 | 0.99 |
| Postprandial Glucose (mg/dL) | 121.96 ± 49.14 | 154.5 ± 26.51 | 156.5 ± 56.28 | 0.52 |
| Systolic Blood Pressure (mmHg) | 124.5 ± 14.93 | 123.98 ± 12.52 | 122.54 ± 14.13 | 0.60 |
| Diastolic Blood Pressure (mmHg) | 81.16 ± 9.96 | 81.28 ± 9.28 | 83.01 ± 9.20 | 0.32 |
| Vitamin D (ng/mL) | 46.71 ± 17.52 | 46.48 ± 17.57 | 42.39 ± 17.17 | 0.16 |

**Supplementary table 5: Association of FOXO3 (Ars13217795) with the following variables**

| **Variables** | **Genotype 1** | **Genotype 2** | **Genotype 3** | **p-value** |
| --- | --- | --- | --- | --- |
| Height (cm) | 162.17 ± 7.59 | 161.51 ± 7.87 | 162.01 ± 8.72 | 0.79 |
| Weight (kg) | 71.39 ± 13.07 | 71.54 ± 12.54 | 70.76 ± 13.40 | 0.90 |
| BMI (kg/m²) | 27.08 ± 4.27 | 27.41 ± 4.40 | 26.92 ± 4.47 | 0.68 |
| Waist Circumference (cm) | 97.23 ± 8.13 | 95.68 ± 9.04 | 95.96 ± 9.10 | 0.37 |
| Hip Circumference (cm) | 99.43 ± 7.04 | 98.41 ± 6.79 | 97.48 ± 7.51 | 0.16 |
| Waist-to-Hip Ratio (WHR) | 0.60 ± 0.05 | 0.59 ± 0.05 | 0.59 ± 0.05 | 0.57 |
| Waist-to-Height Ratio (WHRT) | 0.97 ± 0.06 | 0.97 ± 0.07 | 0.98 ± 0.06 | 0.43 |
| Fasting Blood Glucose (mg/dL) | 109.72 ± 7.70 | 111.07 ± 7.71 | 110.66 ± 7.65 | 0.39 |
| Postprandial Glucose (mg/dL) | 136.53 ± 45.48 | 127.05 ± 50.69 | 156.60 ± 56.28 | 0.78 |
| Systolic Blood Pressure (mmHg) | 123.98 ± 15.25 | 124.52 ± 12.58 | 122.43 ± 13.69 | 0.52 |
| Diastolic Blood Pressure (mmHg) | 80.13 ± 9.83 | 82.12 ± 9.49 | 82.76 ± 8.97 | 0.12 |
| Vitamin D (ng/mL) | 45.60 ± 17.96 | 47.73 ± 17.01 | 41.99 ± 17.31 | **0.04*** |

**Supplementary table 6: Association of SIRT1 with the following variables**

| **Variables** | **Genotype 1** | **Genotype 2** | **Genotype 3** | **p-value** |
| --- | --- | --- | --- | --- |
| Height (cm) | 162.54±7.68 | 161.39±7.66 | 161.78±8.98 | 0.53 |
| Weight (kg) | 72.73±13.75 | 70.94±12.55 | 70.09±12.43 | 0.34 |
| BMI (kg/m²) | 27.50±4.84 | 27.18±4.11 | 26.76±4.22 | 0.51 |
| Waist Circumference (cm) | 98.3±8.85 | 95.31±8.65 | 95.29±8.63 | 0.01 |
| Hip Circumference (cm) | 98.97±7.66 | 98.64±6.68 | 97.55±7.03 | 0.36 |
| Waist-to-Hip Ratio (WHR) | 0.60±0.06 | 0.59±0.05 | 0.59±0.05 | 0.08 |
| Waist-to-Height Ratio (WHRT) | 0.99±0.07 | 0.96±0.07 | 0.97±0.06 | 0.006 |
| Fasting Blood Glucose (mg/dL) | 109.89±7.69 | 111.46±7.87 | 109.79±7.29 | 0.16 |
| Postprandial Glucose (mg/dL) | 129.65±53.15 | 154.5±26.51 | 140.17±56.37 | 0.80 |
| Systolic Blood Pressure (mmHg) | 124.23±14.53 | 124.17±12.85 | 122.56±14.27 | 0.64 |
| Diastolic Blood Pressure (mmHg) | 81.90±10.05 | 81.29±9.58 | 82.10±8.68 | 0.79 |
| Vitamin D (ng/mL) | 45.30±17.81 | 47.08±17.13 | 43.05±17.63 | 0.23 |

**Supplementary table 7: Association of MSTN A55T with the following variables**

| **Variables** | **Genotype 1** | **Genotype 2** | **Genotype 3** | **p-value** |
| --- | --- | --- | --- | --- |
| Height (cm) | 161.98±7.45 | 162.06±7.89 | 161.37±8.84 | 0.80 |
| Weight (kg) | 71.42±13.76 | 71.37±12.44 | 70.99±12.76 | 0.96 |
| BMI (kg/m²) | 27.13±4.44 | 27.13±4.08 | 27.29±4.76 | 0.95 |
| Waist Circumference (cm) | 97.21±8.54 | 95.87±8.68 | 95.69±9.24 | 0.39 |
| Hip Circumference (cm) | 99.07±7.11 | 98.42±6.71 | 97.84±7.63 | 0.48 |
| Waist-to-Hip Ratio (WHR) | 0.60±0.05 | 0.59±0.05 | 0.59±0.06 | 0.49 |
| Waist-to-Height Ratio (WHRT) | 0.97±0.06 | 0.97±0.07 | 0.97±0.06 | 0.77 |
| Fasting Blood Glucose (mg/dL) | 109.80±7.69 | 110.94±7.53 | 110.78±7.90 | 0.49 |
| Postprandial Glucose (mg/dL) | 138.88±50.45 | 143.85±26.94 | 140.17±56.37 | 0.99 |
| Systolic Blood Pressure (mmHg) | 123.95±14.89 | 124.95±12.41 | 121.79±14.22 | 0.23 |
| Diastolic Blood Pressure (mmHg) | 80.35±9.85 | 82.36±9.67 | 82.16±8.70 | 0.22 |
| Vitamin D (ng/mL) | 46.53±17.75 | 46.98±17.47 | 42.06±16.96 | 0.08 |

**Supplementary table 8: Association of MSTN K153R with the following variables:**

| **Variables** | **Genotype 1** | **Genotype 2** | **Genotype 3** | **p-value** |
| --- | --- | --- | --- | --- |
| Height (cm) | 162.41±7.74 | 161.08±7.67 | 162.44±8.80 | 0.31 |
| Weight (kg) | 71.79±13.00 | 70.89±12.47 | 71.34±13.62 | 0.86 |
| BMI (kg/m²) | 27.18±4.43 | 27.29±4.30 | 26.99±4.47 | 0.87 |
| Waist Circumference (cm) | 97.51±8.27 | 95.82±9.29 | 95.47±8.48 | 0.21 |
| Hip Circumference (cm) | 99.28±6.86 | 98.43±7.08 | 97.62±7.32 | 0.27 |
| Waist-to-Hip Ratio (WHR) | 0.60±0.05 | 0.59±0.05 | 0.58±0.05 | 0.30 |
| Waist-to-Height Ratio (WHRT) | 0.98±0.06 | 0.97±0.07 | 0.97±0.06 | 0.52 |
| Fasting Blood Glucose (mg/dL) | 109.33±7.30 | 111.37±7.82 | 110.58±7.80 | 0.12 |
| Postprandial Glucose (mg/dL) | 129.65±53.15 | 126.3±35.87 | 161.32±46.95 | 0.55 |
| Systolic Blood Pressure (mmHg) | 123.48±15.05 | 124.35±12.84 | 123.2±13.70 | 0.79 |
| Diastolic Blood Pressure (mmHg) | 80.4±9.97 | 82.00±9.69 | 82.62±8.52 | 0.23 |
| Vitamin D (ng/mL) | 46.20±17.84 | 45.96±17.25 | 43.98±17.60 | 0.62 |

**Supplementary table 9: Association of VDR (BsmI) with the following variables**

| **Variables** | **Genotype 1** | **Genotype 2** | **Genotype 3** | **p-value** |
| --- | --- | --- | --- | --- |
| Height (cm) | 162.24±7.79 | 161.33±7.76 | 162.18±8.65 | 0.61 |
| Weight (kg) | 71.33±13.34 | 71.71±13.17 | 70.58±12.10 | 0.81 |
| BMI (kg/m²) | 27.01±4.18 | 27.52±4.56 | 26.84±4.31 | 0.46 |
| Waist Circumference (cm) | 97.06±7.94 | 95.96±9.37 | 95.70±8.85 | 0.49 |
| Hip Circumference (cm) | 98.82±6.77 | 98.91±6.91 | 97.40±7.65 | 0.23 |
| Waist-to-Hip Ratio (WHR) | 0.59±0.05 | 0.59±0.05 | 0.59±0.06 | 0.64 |
| Waist-to-Height Ratio (WHRT) | 0.98±0.06 | 0.96±0.07 | 0.97±0.06 | 0.29 |
| Fasting Blood Glucose (mg/dL) | 109.90±7.72 | 110.84±7.54 | 110.85±7.91 | 0.58 |
| Postprandial Glucose (mg/dL) | 128.68±46.08 | 127.05±50.69 | 161.32±46.95 | 0.55 |
| Systolic Blood Pressure (mmHg) | 123.79±14.87 | 124.15±12.77 | 123.20±13.91 | 0.87 |
| Diastolic Blood Pressure (mmHg) | 80.57±9.77 | 81.94±9.41 | 82.56±9.27 | 0.31 |
| Vitamin D (ng/mL) | 46.19±18.38 | 47.48±17.17 | 41.75±16.52 | 0.04 |

**Supplementary table 10: Association of VDR (Taql) with the following variables**

| **Variables** | **Genotype 1** | **Genotype 2** | **Genotype 3** | **p-value** |
| --- | --- | --- | --- | --- |
| Height (cm) | 162.19±7.47 | 161.24±7.78 | 162.43±8.97 | 0.48 |
| Weight (kg) | 71.31±12.72 | 71.64±13.27 | 70.65±12.66 | 0.85 |
| BMI (kg/m²) | 27.05±4.13 | 27.53±4.66 | 26.74±4.16 | 0.39 |
| Waist Circumference (cm) | 97.52±7.94 | 95.74±9.48 | 95.52±8.50 | 0.19 |
| Hip Circumference (cm) | 99.03±6.71 | 98.81±7.21 | 97.23±7.24 | 0.16 |
| Waist-to-Hip Ratio (WHR) | 0.60±0.05 | 0.59±0.06 | 0.58±0.05 | 0.28 |
| Waist-to-Height Ratio (WHRT) | 0.98±0.06 | 0.96±0.07 | 0.98±0.06 | 0.10 |
| Fasting Blood Glucose (mg/dL) | 109.68±7.50 | 111.3±7.8 | 110.34±7.70 | 0.25 |
| Postprandial Glucose (mg/dL) | 121.96±49.14 | 154.5±26.51 | 156.5±56.28 | 0.52 |
| Systolic Blood Pressure (mmHg) | 124.10±15.32 | 124.15±12.68 | 122.79±13.53 | 0.73 |
| Diastolic Blood Pressure (mmHg) | 80.87±9.91 | 81.63±9.53 | 82.73±8.91 | 0.39 |
| Vitamin D (ng/mL) | 46.72±17.93 | 46.43±17.26 | 42.55±17.20 | 0.18 |
